# Building connectomes using diffusion MRI: why, how and but

**DOI:** 10.1002/nbm.3752

**Published:** 2017-06-27

**Authors:** Stamatios N. Sotiropoulos, Andrew Zalesky

**Affiliations:** ^1^ Centre for Functional MRI of the Brain (FMRIB), Nuffield Department of Clinical Neurosciences University of Oxford Oxford UK; ^2^ Sir Peter Mansfield Imaging Centre, School of Medicine University of Nottingham Nottingham UK; ^3^ Melbourne Neuropsychiatry Centre and Melbourne School of Engineering University of Melbourne Victoria Australia

**Keywords:** brain network, connections, parcellation, tracers, tractography, white matter fibers

## Abstract

Why has diffusion MRI become a principal modality for mapping connectomes *in vivo*? How do different image acquisition parameters, fiber tracking algorithms and other methodological choices affect connectome estimation? What are the main factors that dictate the success and failure of connectome reconstruction? These are some of the key questions that we aim to address in this review. We provide an overview of the key methods that can be used to estimate the nodes and edges of macroscale connectomes, and we discuss open problems and inherent limitations. We argue that diffusion MRI‐based connectome mapping methods are still in their infancy and caution against blind application of deep white matter tractography due to the challenges inherent to connectome reconstruction. We review a number of studies that provide evidence of useful microstructural and network properties that can be extracted in various independent and biologically relevant contexts. Finally, we highlight some of the key deficiencies of current macroscale connectome mapping methodologies and motivate future developments.

## 1 INTRODUCTION

Functional integration, the interaction and information transfer between different subunits in the brain, is mediated in part through white matter connections.^1^ The formation of these fiber pathways is guided by genetic, but also environmental, factors. During the early phases of development, an initial over‐production of synapses is followed by pruning of the redundant connections in response to first life experiences.^2^ The continuous maturation and myelination of white matter from the first months of life and through to adulthood reflects learning and interactions with external stimuli.

This experience‐dependent molding of brain connectivity^3^ sheds light on the functional relevance of white matter pathways. Anatomical connections constrain neural computations. In fact, the pattern of anatomical connections a brain region has with other regions can predict, to a certain extent, the function of that region at a systems level.^4^, ^5^ This notion of connectivity fingerprinting and its functional implications has increased interest in studying connections and structural organization.^6^ The term *connectome*, proposed roughly 10 years ago,^7^, ^8^ describes a comprehensive network map of extrinsic connections between functionally specialized brain regions. Ideally, such a map contains not only a list of connected areas, but also the relative strength and directionality of each connection.^8^ Connectomics has the potential to reveal new insights into the principles that guide how different functional subunits are arranged and influence one another,^9^ as well as how these processes are perturbed in pathological brain conditions.^10^

Invasive approaches to map brain connections have existed for many decades.^11^ At the microscale, techniques such as automated histological staining,^12^, ^13^ serial electron microscopy^14^ and 3D fluorescence imaging^15^ allow more data to be collected and processed nowadays with less labor‐intensive methods and fewer imaging distortions. However, the small field of view of microscopy techniques limits their applicability to small model species, such as the nematode C. elegans,^16^ and mapping exquisite details of small tissue segments in larger species. At the mesoscale, chemical tracers are considered to be the gold standard for mapping longer‐range white matter connections as they allow very high measurement accuracy and detail. In fact, the majority of our knowledge about white matter organization has been obtained through tracer studies (see Jbabdi et al^17^ for a review), and macroscopic connectome matrices for different animals and scales have been obtained (for instance References 18, 19, 20, 21, 22, 23).

Non‐invasive imaging techniques offer an alternative modality for connectome reconstruction at the macroscale in living humans.^17^ Diffusion MRI (dMRI) and tractography techniques (see other review papers in this issue) have been successfully used for many years now to reconstruct the trajectories and estimate microstructural properties of fiber bundles in white matter.^24^ In comparison to invasive approaches, these methods are indirect: they do not explicitly measure the quantity of interest, but rather rely on models and inference. For this reason, they are error prone and the results can be more difficult to quantify compared with those of corresponding invasive methods.^25^ They also offer substantially lower spatial resolution than chemical tracers and microscopic techniques and they cannot estimate the directionality of connections. However, *in vivo* mapping of connections in humans offers the potential of considerable advantages^6^: (i) many connections in many subjects can be studied simultaneously; (ii) structural connections can be mapped along with function, behavior and genetics and (iii) changes in connections with development, aging or pathology can be probed.

In this review we consider existing methodologies for mapping the connectome using dMRI. We discuss the impact on connectome reconstruction of different image acquisition parameters, fiber tracking algorithms and other methodological choices. We highlight comparative and validation studies that provide evidence for the potential of these methods but also reveal their deficiencies. Furthermore, we consider features of white matter connectivity that are inherently difficult to reconstruct with existing approaches. Such features impose limits on the biological specificity of dMRI‐derived quantities and motivate new developments and shifts in current connectivity mapping paradigms.

## 2 BUILDING A CONNECTOME

*In vivo* MRI methods provide a *macroscopic* view of the connectome. Connections that can be reconstructed with dMRI and tractography are the extrinsic *
Extrinsic^4^, ^9^ connections that traverse white matter include local U‐fibers (up to ~30 mm in length) connecting neighboring regions and long‐range fascicles connecting remote regions within or between hemispheres. Intrinsic connections are horizontal and intra‐cortical (<3 mm in length). It is estimated that the number of intrinsic connections (~10^11^ fibers) is an order of magnitude larger than the number of U‐fibers (~10^10^). The number of U‐fibers is an order of magnitude larger than the number of long‐range connections (~10^9^ fibers).^26^ Although there are considerably fewer extrinsic connections than intrinsic ones, extrinsic connections provide the network links necessary to achieve functional integration. pathways between regions that traverse white matter. Although these constitute only a small fraction (~10%) of the total number of neuronal connections^26^ (the others are intrinsic, intra‐cortical), they are important to study in order to understand the brain at the level of a system and they are characterized by considerable complexity (see Van Essen et al^27^ for a review of these complex features).

**Figure 1:**
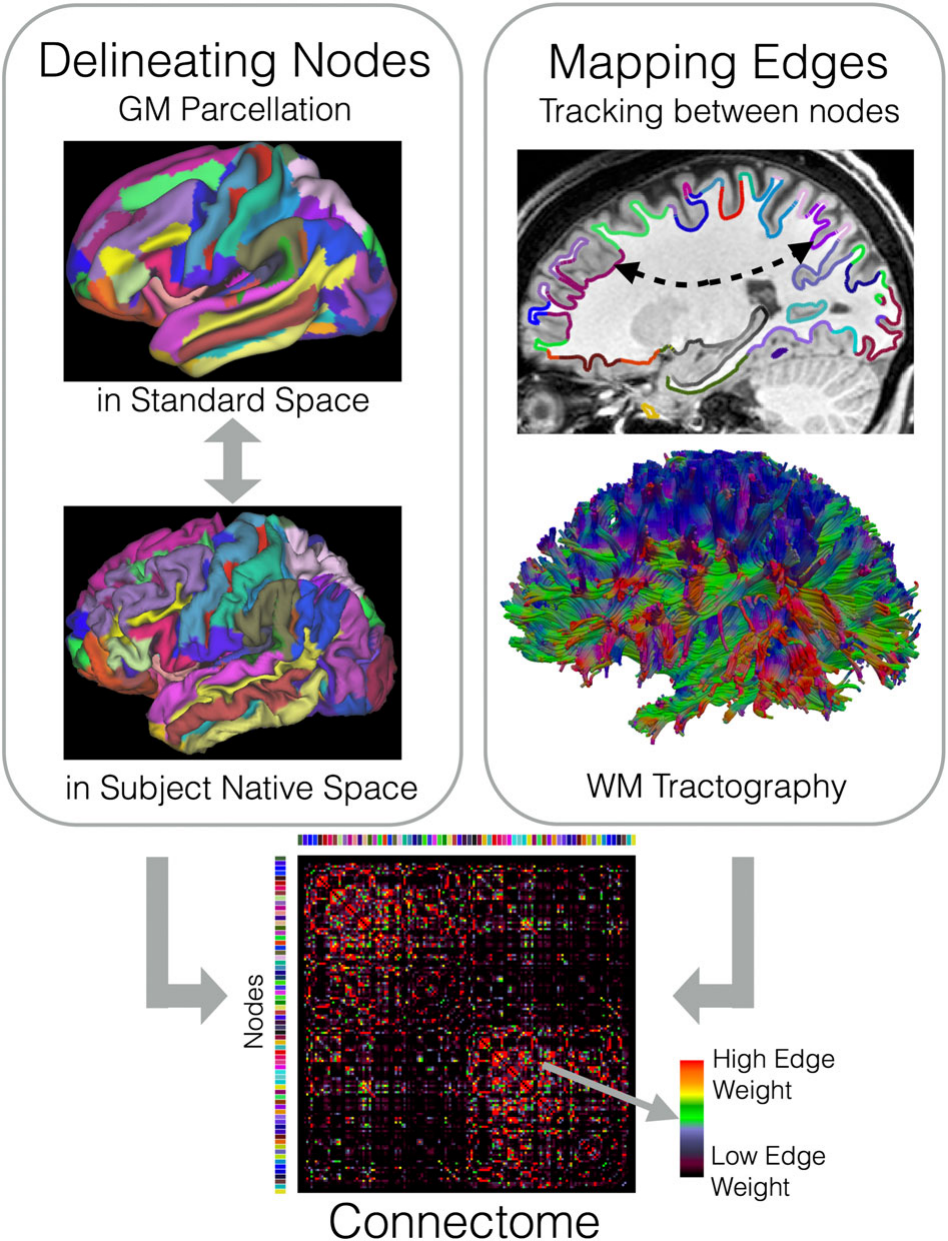
Generating a macroscale connectome involves estimating its nodes and edges. The nodes are typically gray matter (GM) regions (cortical and subcortical) defined either geometrically, functionally or cyto/myelo‐architectonically. The edges represent connection pathways between the nodes. dMRI and tractography can probe these white matter (WM) connections and estimate relative weights

Inferring a *macro‐*connectome from dMRI images is a very challenging task and a field of active research. We divide connectome mapping into two tasks: node delineation and edge mapping (Figure 1). Nodes represent spatially distinct cortical and subcortical gray matter regions, while edges represent the white matter fiber bundles that interconnect pairs of regions.

### 2.1 Node delineation

Specifying a parcellation scheme that subdivides cortical and subcortical gray matter into discrete, spatially contiguous parcels is not straightforward.^28^ Architectonic atlases provide the simplest and perhaps most commonly used approach. Many such atlases are available (see Reference 29 for a review) and they can be registered to an individual brain to ensure that nodes between subjects are matched with respect to average size, geometry and location. However, architectonic and other template‐based atlases do not capture variation between individuals in regional functional boundaries and thus make the simplifying assumption that a common parcellation is representative of all individuals. For instance, the AAL^30^ and Harvard‐Oxford^31^ atlases are based on anatomical landmarks, while the parcellation of the Talairach Daemon^32^ and the Juelich atlas^33^ are based on cytoarchitectonic features from post‐mortem brains.

Data‐driven parcellations offer an individually customized alternative. Data from task‐based or resting‐state functional MRI can be used to define areas with homogeneous features across a population comprising hundreds of subjects.^34^, ^35^, ^36^, ^37^, ^38^ In this way, regions are delineated based on functional properties that are specific to the individuals for whom connectomes are to be mapped. Methods for defining functional boundaries are many and varied, with the most recent utilizing multiple modalities and unique measurements. For instance, Reference 39 uses multiple features simultaneously, such as cortical folding, myelin content and resting‐state and task‐based activity, to identify a functionally relevant and population‐specific parcellation, which respects individual variability. In Reference 40, the authors use structural, functional and behavioral features to identify a parcellation consistent across various domains.

#### 2.1.1 Limitations and open problems

dMRI and tractography methods can be also used to extract connection patterns and probe functional boundaries.^4^, ^9^ This has been shown with controlled studies for a large number of regions in the cortex and subcortex (for instance References 41, 42, 43, 44; see also Section 3.2). However, current limitations in structural connectome estimation, as we explore throughout this review, can limit the accuracy, interpretability and generalizability of such approaches for node delineation.

While parcellating the cortex based on functional homogeneity is an attractive alternative to node delineation, it also suffers from methodological caveats.^45^ Furthermore, a number of conceptual problems exist with respect to the definition of areal boundaries.^46^ Here, we briefly review some of these considerations.

##### 2.1.1.1 Individual variability

Individual variability in brain function and structure complicates the interpretability of population‐level parcellations. Well‐characterized areas, such as V1, can vary twofold in areal size across subjects.^46^, ^47^ The relationship between functional boundaries and cortical folding is also highly variable. Areas associated with high‐order function exhibit more variability across subjects in folding patterns than primary areas, where the folds can be reasonable predictors of the boundaries.^48^ Therefore, a group‐level parcellation template cannot capture subtle yet important variations between individuals. Registration frameworks that align functional features rather than simply matching geometry or cortical folding could offer a way to approach this problem.^49^

##### 2.1.1.2 Within‐region heterogeneity

Different patterns can characterize the functional and topographical organization of a region. For instance, different within‐area topographical organizations can underlie different functions of the same region.^50^ V1 and V2 provide such examples, with their central and peripheral subregions exhibiting different relationships with other regions of the cortex.^46^ This within‐area heterogeneity, along with individual variability, makes delineation of boundaries very challenging. A potential solution is to define smooth transitions and fuzzy boundaries between regions rather than binary parcellations. However, this fuzziness should reflect uncertainty in functional features rather than data noise, folding differences or alignment errors.

##### 2.1.1.3 Scale and number of nodes

In many studies, the coarseness of gray matter subdivisions is a relatively arbitrary choice. Principled data‐driven methods to perform model selection and define an optimal number of nodes exist for parcellations based on functional data.^37^ However, these methods may be conflicted by the trade‐off between true functional relevance and discriminative power of the available data. For instance, using fMRI versus MEG data is accompanied by different limitations in spatial and temporal resolution, which can influence model selection. For this reason, some investigators have resorted to multi‐scale schemes to ensure findings are generalizable and not sensitive to a given parcellation resolution.^51^, ^52^

The coarseness of a node parcellation influences the process of mapping white matter connections. In practice, edge mapping and node delineation are performed independently and the outputs of these two processes are only combined when mapping a connectivity matrix. Parcellations with fewer regions tend to give more reproducible and “smooth” mappings than finer ones, whereas more detailed parcellations in principle preserve more details.^53^, ^54^ Low‐ to mid‐scale parcellations (of the order of tens to a few hundred regions) have been shown to increase agreement of dMRI‐estimated connectomes with tracers in the mouse^55^, ^56^ and monkey brain^57^ when compared with results from finer subdivisions.

In summary, methods for delineating connectome nodes are many and varied. The number of nodes and specific nodal parcellation that are best suited to a given application are usually not obvious. Parcellations that are informed by brain anatomy and/or functional specialization have gained significant traction in the field. In the future, parcellation strategies should be developed that capture individual variability. Unlike the microscale, where the parallel between nodes and neurons is obvious, defining nodes at the macroscale is less clear. It is therefore important to assess the consistency of results for a coarse anatomical parcellation as well as a finer parcellation that is delineated functionally or perhaps randomly. Inconsistencies that emerge between these two scales can potentially shed light on the nature of a particular finding.

### 2.2 Mapping edges

Once the nodes of a connectome have been defined, tractography can be used to estimate edges—the connecting paths between pairs of regions. While other indirect methods exist,^17^, ^58^ dMRI‐based tractography is the only method that allows localization of white matter bundles *in vivo* (see References^59^, ^60^ and papers in this special issue for reviews). Axonal fiber bundles are organized coherently such that water diffusion occurs preferentially along the orientations of least hindrance, which are typically parallel to the fibers. In contrast, diffusion is maximally hindered in the perpendicular direction. The preferred diffusion orientations (PDOs) can be indirectly mapped to fiber orientations at a voxel‐wise level (see References^61^, ^62^ and papers in this special issue for reviews). Tractography approaches then integrate the voxel‐wise information at a global scale and propagate curves that are maximally tangential to the local PDOs.^63^ These curves provide estimates of the white matter bundles.^64^

There are a plethora of methods for mapping fiber orientations and for curve propagation. The diffusion tensor model^65^ is the simplest approach and provides a unimodal approximation to the underlying fiber configurations. A more accurate model in the case of complex fiber patterns is the fiber orientation density function (fODF), which characterizes the fiber distribution in each voxel. Deconvolution methods, parametric^42^, ^66^, ^67^, ^68^ or non‐parametric,^69^, ^70^, ^71^ *q*‐ball imaging^72^ and diffusion spectrum imaging^73^ are some of the popular methods that can provide estimates of the fODF †
Strictly speaking, *q*‐ball and diffusion spectrum imaging provide an estimate of the diffusion ODF, a blurred version of the fODF. and a discrete number of crossing orientations in each voxel. The importance in estimating crossings and the maturity of deconvolution methods have been shown in many instances for tracking deep white matter ‡
We use the term “deep white matter” to denote all white matter under the white‐gray matter boundary. This is in contrast to white matter at the boundary and above, where connection terminations occur. (e.g. References 73, 74, 75, 76, 77, 78). A recent study showed benefits of considering fODFs for tracking into the transition from white matter to gray matter.^79^

Tractography methods can be grouped into two categories: (i) local approaches,^63^, ^80^, ^81^, ^82^ which in a greedy, step‐by‐step fashion propagate curves (or streamlines) that are tangent to vector fields extracted from the fODFs; (ii) global methods (for instance References 83, 84, 85, 86, 87, 88, 89), which estimate paths that are optimal according to a global criterion. Such paths are not necessarily tangent at every point of their route to the local fODF/vector fields. In principle, they are less susceptible to local errors. Local methods have been by far the most popular and applied approaches. Global methods offer a promising alternative,^77^, ^90^ but they require further validation, and they can be more cumbersome and computationally demanding. Global methods have been tested mostly for deep white matter tracking, and thus the extent to which they share or solve the limitations inherent to local methods (see next section) is yet to be explored.

Local streamline methods can be further subdivided into deterministic and probabilistic, depending on whether they perform a deterministic or stochastic estimation. Deterministic methods^63^, ^81^ provide a point estimate of the path of least hindrance to diffusion between two points. Probabilistic methods^42^, ^82^ estimate a spatial distribution for this path. They estimate the uncertainty around the local fODF peaks (through parametric or non‐parametric inference)^42^, ^82^, ^91^ and account for this uncertainty to obtain the path distribution (peak‐based probabilistic methods). A variant of these approaches uses samples from the whole fODF to obtain the distribution (whole‐fODF probabilistic methods; see Jeurissen et al^92^ for an illustration of this difference).

Several studies have evaluated the test–retest reliability of these methods for connectome reconstruction.^93^, ^94^, ^95^ Estimates derived from probabilistic tractography generally show greater connectome reproducibility than deterministic methods, reduce the effect of residual spatial misalignment errors and potentially improve some of the statistical properties of the sampled paths (i.e. normality). At the same time, they can be to the detriment of connectome specificity and accuracy.^96^ Probabilistic tractography yields greater spatial dispersion in streamline trajectories, which may lead to more spurious connections (particularly for whole‐fODF sampling methods^97^). On the other hand, connectomes derived from deterministic tractography generally comprise fewer connections, but results show substantially greater variation within and across individuals (particularly in data with low angular resolution or low signal‐to‐noise ratio (SNR)). It is unclear what proportion of this variation represents genuine anatomical variation between individuals, as opposed to noise due to a poor fitting local fiber orientation model, tractography errors or residual misalignment between the streamlines and regional brain atlas.

The seeding strategy also has an effect on finding the edges of a connectome.^98^ Streamlines are typically initiated from all white matter and the streamlines intersecting pairs of nodes are mapped to the respective edge.^8^ This “brute force” approach was originally suggested to be more sensitive to the detection of long white matter bundles.^99^ An alternative is to seed from the boundary between white and gray matter.^100^, ^101^ Boundary seeding has been recently shown to provide smaller biases of dMRI estimates when compared with biological ground truths (for instance less gyral bias, better predictions of path length distributions, slightly better sensitivity versus specificity performance).^23^, ^102^

#### 2.2.1 Limitations and open problems

##### Diffusion‐to‐axon mapping is ill posed

Finding connections is based on a mapping from water diffusion to fiber orientations, which is inevitably required due to the indirect nature of dMRI. Such inference is in general an ill‐posed problem,^60^, ^61^ but the problem becomes identifiable using approximations and assumptions. Improving data quality and estimation methods to reduce potential errors arising from these assumptions has therefore been at the heart of dMRI research.

**Figure 2:**
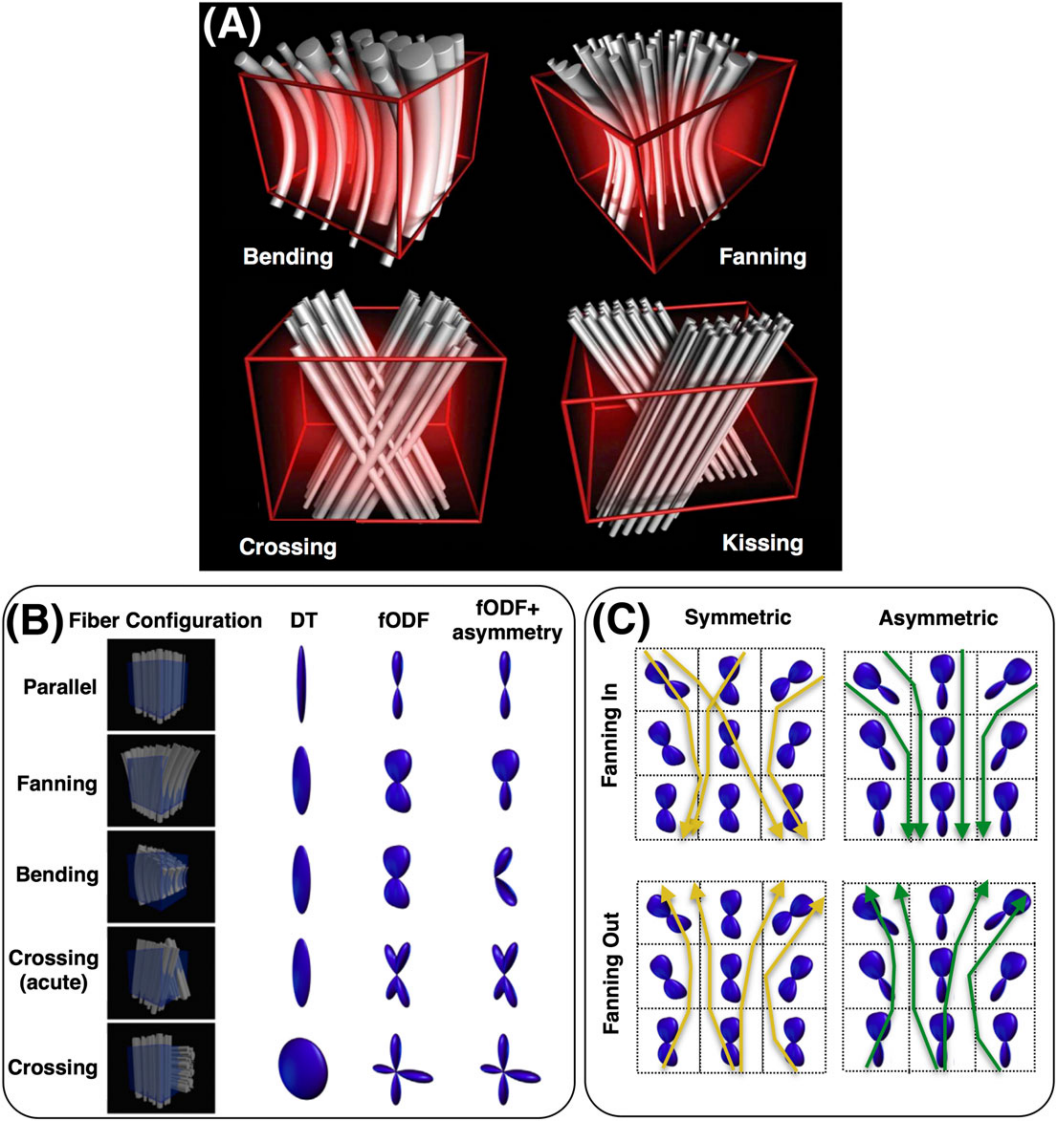
A, Examples of complex fiber patterns that can result in very similar within‐voxel dMRI signals. Reproduced with permission from Tournier et al^61^. B, The estimated diffusion tensor (DT) and fiber orientation density function (fODF) are shown for different patterns. The last column shows an asymmetric fODF, which cannot be estimated in practice using voxel‐wise data alone, given that the voxel‐wise dMRI signal is inherently antipodally symmetric. Modified with permission from Seunarine et al^62^. C, Hypothetical example showing the effect of asymmetric fODFs on fiber tracking within fanning geometries. The asymmetry in voxel‐wise estimates provides asymmetry in propagation, with the aim of reducing false positives in the case of tracking converging (fanning in) geometries. Recreated from Jbabdi et al^60^

MRI voxels (even at high resolution) are too large to enable the resolution of axons. Thousands of axons coexist within the volume occupied by an imaging voxel. The measured macroscopic signal is therefore considerably far from the scale of interest. As a result, different ground‐truth fiber patterns can lead to very similar signal profiles within a voxel. An example is shown in Figure 2A. Voxel‐wise fODF estimation does not have the information to differentiate between these patterns, as they correspond to similar signal profiles. The current approaches rely on approximations and typically assume that patterns with fiber orientation dispersion provide evidence for crossing fibers. Looking for and using discrete local maxima in these fODFs (when in reality there is a fanning or a sharp bending pattern) can lead to false positives and negatives.^60^ Efforts have been made to extract continuous features (other than the maxima) from the fODF and go beyond crossing fibers.^66^, ^103^, ^104^, ^105^, ^106^ However, taking advantage of this information in tractography is not straightforward and only recent frameworks explore such integration.^109^, ^110^, ^112^

Another factor that compounds the difficulty in identifying local fiber orientations is the axial symmetry of the diffusion signal. Diffusion in opposing directions will give rise to the same measurement. As a result, voxel‐wise fODF estimates are antipodally symmetric as well, even if the ground‐truth patterns are not. Figure 2B,C shows the errors caused by ignoring this asymmetry when tracking diverging and converging bundles.

These inherent limitations make tractography methods very prone to errors. Imposing anatomical constraints^56^, ^101^ and/or tractography filtering^107^, ^108^ offers ways to reduce false connections in principle. However, these do not solve the problem of missing connections (false negatives), and caution is needed as filtering can generate spurious between‐group differences due to the effective re‐distribution of streamlines. These limitations highlight the need for new paradigms. For instance, inferring asymmetric fODFs is possible by considering neighborhoods of voxels rather than individual voxels.^109^, ^110^, ^111^, ^112^ Augmenting tracking with microstructure^113^, ^114^, ^115^, ^116^ offers a more robust alternative to orientation‐based tracking, as estimated bundles have structural features preserved along their route rather than orientation alone.

##### Finding terminations is inherently limited

Mapping a connectome demands accurate fiber tracking in deep white matter as well as accurate determination of fiber termination points in grey matter. Identifying fiber termination is inherently difficult with tractography.^60^ In fact, tractography cannot terminate propagation in an unsupervised manner and heuristics need to be used to determine endpoints. This makes termination criteria an important choice and is the reason why anatomically driven rules can improve reliability of results.^100^, ^101^ For instance, crossing the white/gray matter boundary (WGB) multiple times is likely to yield spurious trajectories, and propagation within the cortex may be prone to greater errors due to low anisotropy in grey matter.

Recovering laminar organization of connectivity is an example where finding terminations with current dMRI technology is impossible. Different layers in the cortex may preferentially connect to different regions.^17^, ^117^ Layer‐specific orientation information has been shown with extremely high spatial resolution imaging of *ex vivo* tissue.^118^ However, even if this information was available *in vivo* (for instance with some variant of the approach proposed by Barazany et al^119^), termination points to different layers would not be estimable using tractography,^60^ as the algorithms are insensitive to synaptic endpoints. Similar problems exist with subcortical/cerebellar nuclei, within which tractography cannot find synaptic termination locations. A difference, however, is that the inherently higher anisotropy *within* major subcortical structures allows topographical organization and connection patterns to be probed within their volumes (for instance see References^42^, ^120^ or Figure S3 in Glasser et al^121^). In the case of cortex, due to low anisotropy, contrast and the inherent resolution limits, we are mostly sensitive to connectional patterns *along* the cortical sheet (WGB) (see Section 3.2 for examples).

**Figure 3:**
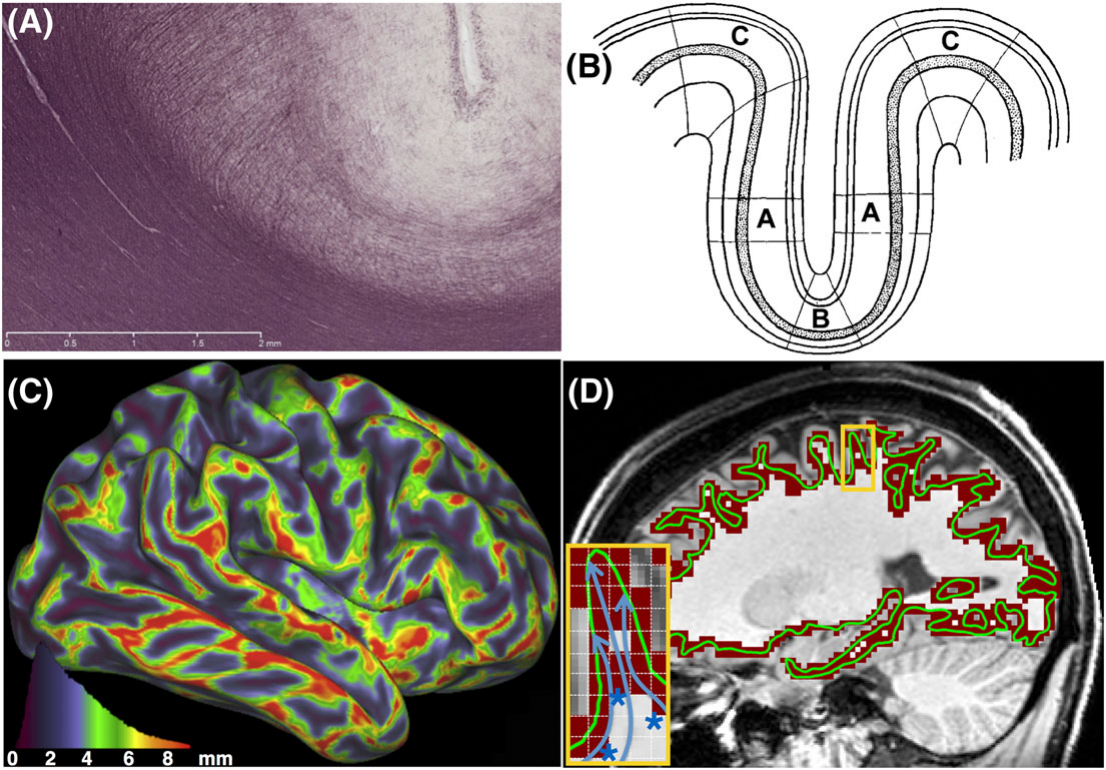
A, A myelin‐stained slice showing fiber patterns in the cingulate sulcus of an 18‐month‐old macaque. The major fiber orientations run parallel to the WGB. B, Folding‐related effects on radial axes and cortical thickness. The cortex is thinnest at the sulcal fundi (region B) and thickest at the gyral crowns (region C). C, Expected gyral bias based on estimated surfaces and cortical thickness. The cortical volume (mm^3^) per mm^2^ surface area of the WGB surface is displayed on an inflated human brain (right hemisphere). A‐C are adapted with permission from Van Essen et al^27^. D, Defining boundaries using surfaces versus volumes. The WGB is shown as a surface (green outline) comprising vertices that are 2 mm apart. The same boundary has been projected onto a volume (dark red region) comprising voxels that are 2 mm apart. The projection was performed using the nearest voxel for each vertex of the 3D surface. Both boundaries are superimposed on a high‐resolution T1w image (sagittal view). The inset (yellow outline) shows a magnified view of a gyrus, as described by the two methods. The volumetric description lacks specificity and precludes differentiation of termination points of incoming pathways (blue curves) at different locations of the boundary. For instance, locations at the sulcal fundi (blue asterisks) can be perceived as termination points of the incoming streamlines, even if these are directed towards the gyral walls and crown

In the cortex, another obstacle that biases the estimation of termination points is the presence of superficial white matter fibers, such as the U‐fibers that run parallel to the WGB^122^ (see myelin‐stained fibers at a sulcal fundus in Figure 3A). The density of these fibers is higher at the sulcal fundi, meaning that dMRI‐estimated fiber orientations are mostly parallel to the sulcal surface. It is therefore difficult for tractography to traverse the boundary and escape white matter. This under‐representation of tractography streamlines at the sulci compared with gyri was first described by Van Essen et al^27^ as *gyral bias*. This bias is expected to be more evident for finer parcellation schemes and a large number of nodes, yet it can potentially introduce a confound for coarser parcellations as well, particularly when average curvature and sulcal depth profiles vary considerably across nodes.

### 2.3 Quantifying edges

As described above, tractography can provide an estimate of the trajectories representing fiber bundles. Ideally, a connectome should also include estimates of connection strengths (for instance axonal densities, myelination, diameter). dMRI cannot provide such direct measures,^25^, ^60^ but allows estimation of edge weights that indirectly reflect some of these properties of interest. These range from simple binary values, denoting the presence or absence of an edge, to approximations of biophysical properties of connections, reflecting micro‐ or macro‐structure.

Connection strength is most typically quantified using some function of *streamline counts*, the number of streamlines intersecting a pair of regions. Streamline counts can be enumerated for all pairs of regions to populate the cells of a connectivity matrix.^7^, ^8^ Streamlines that are permitted to propagate within grey matter can intersect more than two distinct regions, in which case they contribute to the streamline count for multiple pairs. Anatomical constraints can be imposed to avoid such scenarios, which terminate streamlines either at the white/gray matter interface or within subcortical volumes.^101^

Streamline counts can be symmetrized, normalized or transformed in various (nonlinear) ways,^85^ which aim to reduce the effect of confounds reflecting algorithmic choices and ensure better consistency across subjects. Power transforms, in particular the logarithm, can be applied to the streamline counts before analysis to achieve normality. Normalization by node sizes^8^, ^123^ can be used to account for volume/area variability in the chosen gray matter parcellation. While it may be that larger brain regions are indeed more strongly connected by virtue of anatomy, a greater number of streamlines is likely to terminate in regions with a larger interface between grey and white matter due to the tractography process.^53^, ^100^ Normalization by row and column sums of the matrix, such as fractional scaling, provide enhanced relative contrast of a particular edge to the rest of the edges that involve any of the two connecting nodes and improves the power to predict tracer‐measured connection strengths using tractography‐derived weights.^23^

Diffusion path probabilities, obtained from probabilistic tractography, reflect normalized conditionals of streamline counts given the orientation model, seeding strategy and termination/counting criteria.^74^, ^82^ As discussed before, due to the stochastic generation of streamlines, probabilistic tracking provides a spatial distribution on the path of least hindrance to diffusion. The relative contrast of path probabilities have been recently shown to correlate with connection strength measured using tracers,^23^ similar to deterministic streamline counts.^124^ However, path probabilities are also confounded by many uninteresting factors (such as path geometry, noise, modeling errors), which make direct interpretations difficult.^25^, ^60^

Alternative metrics that reflect *microstructural* properties along edges can be considered as edge weights. For instance, voxel‐specific measures of anisotropy can be averaged over all voxels traversed by a path that is assigned to a particular pair of nodes.^125^ The resulting tract‐averaged measure thus characterizes the anisotropy of a connectome edge as a whole. Other microstructural measures can be also used,^125^, ^126^ such as axonal myelin content measures derived from images of magnetization transfer ratio.^127^, ^128^ Different weighting functions can be employed for the averaging process to give greater weight to different parts of the tract (e.g., using streamline counts/probabilities in each voxel can give greater weight in the main tract core versus the periphery).

Features that reflect tract *macrostructure* are another possibility for edge quantification. In fact, volume and cross‐sectional area of paths intuitively relate more directly to connection strength than their microstructure counterparts. Close et al^129^ use explicitly the tract volume in a model to parameterize connection paths using spatial basis functions. Extensive simulations illustrate the potential to infer volume as a probe of apparent connection strength, but high computational demands currently limit exploration, and utility in real data is yet to be shown. Smith et al^108^ present a post‐processing filter of streamlines based on a generative model of the data from streamlines (similar in spirit to References^107^, ^130^). The filtered streamline counts are proposed as probes of the cross‐sectional area of the white matter connection underlying an edge. Even if such an interpretation is based on the assumption that relative fODF volume fractions reflect relative axonal densities, the filtering increases the biological relevance of the obtained edge weights.^102^

#### 2.3.1 Limitations and open problems

The major limitation for quantifying edges is that none of the above approaches provide directly an inter‐regional measure of the number of connecting axons, which is a desirable measure of connectivity strength in typical neuroanatomy applications. Tract‐averaged microstructural measures may provide an interpretable biophysical property per edge. However, it is questionable how informative such properties are when treating connectomes as networks, which would require some proxy of connectivity. In a recent study no correlation was found between such microstructural measures and axonal strengths measured by tracers.^124^ On the other hand, functions of streamline counts can be thought to be more relevant in such a network context.^23^ However, factors that reflect data quality, algorithmic choices and inherent limitations bias these measures,^60^ as we discuss below.

Future work should also focus on characterizing the distributional and noise properties of connectivity matrices. Connectivity matrices comprising streamline counts derived from probabilistic tractography are likely to show smoother variations between spatially neighboring node pairs compared with deterministic tractography, as streamline trajectories associated with probabilistic tractography are more spatially dispersed.

##### Distance bias

Streamline counts between distant regions, interconnected by longer tracts, are often smaller than counts between neighboring regions. Algorithmic limitations contribute to this pattern; for instance, longer tracts are more difficult to reconstruct with tractography because streamlines must be propagated for a longer distance and each propagation step provides an opportunity for “wrong turns”.^131^, ^132^ However, connection strengths as measured by tracers follow an exponential decay with connection length, with the majority of connections being short and strong and the long connections being weak, comprising fewer axons.^133^, ^134^ Tracers of course have their own error sources,^17^ but the extent to which the algorithmic distance bias of tractography is biologically specific remains to be explored.

##### Seeding bias

When streamlines are seeded from all of white matter, longer tracts are inevitably sampled more abundantly because they occupy a greater volume than shorter tracts. To compensate for this bias towards long tracts, the streamline count can be normalized by the average length of the streamlines contributing to the count.^135^ However, given that many tracts are sheet‐like and vary considerably in cross‐sectional area and morphology, simple normalization factors such as the streamline length might not adequately correct for the over‐sampling of tracts occupying greater volumes. Filtering of streamlines via generative models that ensure higher fidelity with the data^107^, ^108^, ^130^ is another approach that seems to be more beneficial in this context. Initiating streamlines from the WGB interface is an alternative that overcomes this limitation and can potentially provide more realistic path length distribution,^23^, ^100^, ^102^ but this seeding approach has difficulty in tracing out long fiber bundles.

##### Gyral bias

As described in the previous section, the existence of superficial tangential white matter can lead to under‐representation of tractography streamlines at the sulci compared with gyri,^122^ the so‐called gyral bias.^27^ The bias is further magnified by algorithmic limitations in tractography; sharp turns (needed to capture axons, which can bend at quasi‐right angles^15^) are less preferred than linear trajectories that will lead most of the time to the gyral crowns.

The gyral bias is biologically relevant. The preferential termination of tractography streamlines at gyral crowns agrees with neuro‐anatomical expectations. It is the tractography‐predicted magnitude of the difference for preference towards gyral crowns compared with sulcal fundi that is unrealistic.^27^ Indeed, let us postulate that the number of axons crossing the WGB is constant per unit cortical volume (as we have no evidence to assume otherwise). Cortical folding however induces geometrical differences in different parts of the cortex. Cortex tends to be thickest along gyral crowns and thinnest in sulcal fundi^136^ (Figure 3B), and larger cortical volume corresponds to a unit surface area of the WGB at the crown compared with the fundus. Therefore, even if the number of axons crossing the WGB boundary is roughly the same per unit volume of cortex, the density of axons per unit surface crossing at the gyral crowns will be larger compared with the axon density crossing at the sulcal fundi. Van Essen et al. used cortical thickness measures to compute this expected bias (Figure 3C) and found that it is four to five times less than that predicted by tractography.^27^

New paradigms are needed to address these limitations when tracking close to the WGB. Some very recent studies have taken preliminary steps in this direction. Cottaar et al^137^ explore the relationship of fiber orientations to cortical features using post‐mortem high‐resolution histology to inform generative models for *in vivo* dMRI. St‐Onge et al^138^ impose a strong prior on the fiber orientations within the cortical ribbon and use flow preservation constraints to successfully track into/out of various locations along the WGB. Approaches for neighborhood‐wise tracking, such as those used before for deep white matter,^110^ may also be beneficial for improved estimates of the transition between white and gray matter.

##### Surfaces or volumes?

Boundaries, such as the WGB, can be represented either as volumes or as surface meshes. Surface representations offer a more compact and accurate description of the boundaries at a given resolution.^139^ This can have an effect on the tractography results and their interpretation. For instance, an intermediate‐resolution, voxel‐based representation of the WGB cannot necessarily follow the highly convoluted boundary (Figure 3D) and can mask the gyral bias. As shown in the inset of Figure 3D, the streamlines are frequently unable to reach a voxel representing the gyral crown without first traversing a voxel that represents the sulcal fundus. Depending on the width of a gyrus and on how tractography boundary conditions are imposed, this can artificially increase the visitation frequency to certain sulcal regions (blue asterisks in Figure 3D). However, these frequencies and their spatial pattern may purely reflect resolution‐induced limitations of the voxel‐wise representation. Using surface meshes, such as GIFTI files,^139^ should be more robust to these problems, but the exact differences between results obtained from the two representations remain to be explored.

##### Summary

The reconstruction and mapping of connectome edges is confounded by many limitations and open problems remain to be solved. Reproducible, sensitive and specific measures of white matter connectivity are difficult to obtain with dMRI. Despite these limitations, dMRI enables reconstruction of connectomes, which display biological properties that are consistent with brain networks mapped with alternative modalities. Recent studies explore this directly,^23^, ^102^, ^124^ while a plethora of studies provide indirect evidence in favor of consistency between modalities. We review this evidence in detail in Section 3.

### 2.4 Impact of data quality

So far we have discussed the impact of algorithmic and methodological choices on building connectomes. Another important aspect that affects estimation of dMRI‐derived quantities in general (and therefore connectomes) is data quality. Given the inherent limitations of dMRI, better data can help if developments allow: (i) improvements in diffusion‐to‐axon mapping and (ii) reduction in partial volume. SNR, spatial resolution, angular resolution and angular contrast are some of the data features that can directly influence these factors.^28^ Even if some limitations cannot be directly overcome with better data (see Reveley et al^122^ and some of the conceptual problems described in the previous sections), significant improvements in tracking white matter have been shown.

More specifically, better SNR and/or angular resolution improve precision and accuracy of the mapping from diffusion signal to fiber orientation estimates.^140^ Improved angular contrast allows higher sensitivity in detecting within‐voxel complex fiber patterns,^69^, ^73^, ^76^ which further allows more accurate tractography.^74^ Multiple angular contrasts (i.e. *b* values) give better estimation of partial volume and differentiation of diffusion compartments and further augment accuracy of orientation estimates.^141^, ^142^

Increased spatial resolution reduces partial volume and allows imaging of exquisite details of white matter organization.^143^, ^144^, ^145^ For instance, very high‐resolution dMRI allowed the mapping of axonal and dendritic networks in the hippocampus, whose accuracy was confirmed with tracers. High spatial resolution is also beneficial for better distinguishing tract termination points.^146^ Increasing the resolution by using high field strength improves the estimation of the fiber spreading pattern to the cortex and reduces gyral bias.^147^ In another recent study, improving on all the data quality aspects of dMRI data increased agreement of connectomes estimated in humans using three different modalities.^148^

**Figure 4:**
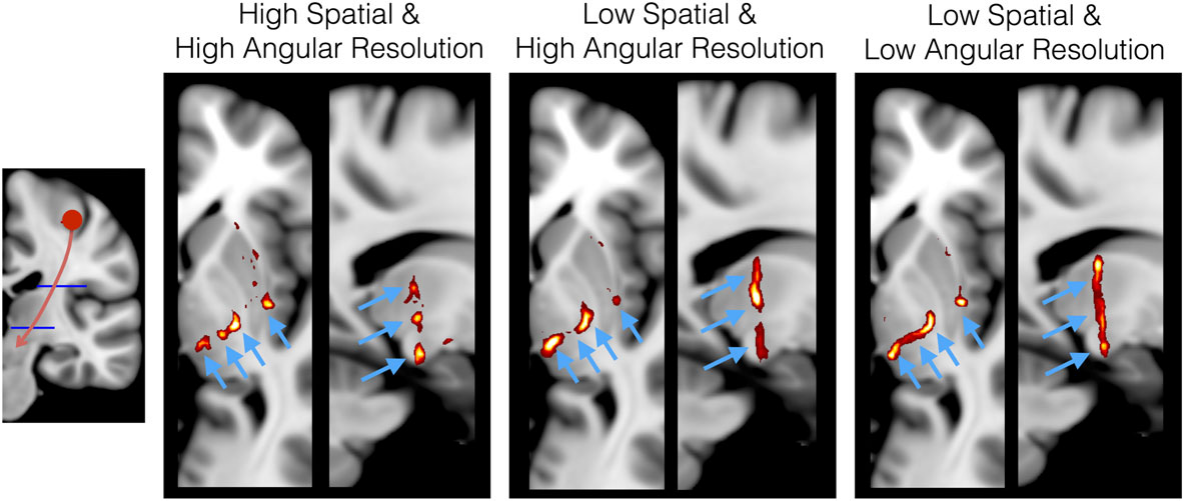
Tractography results from the hand area of the motor cortex for different spatial and angular resolutions. The same subject was scanned at two different spatial resolutions, (1.35 mm)^3^ and (2.5 mm)^3^, using a Siemens Prisma 3 T. The diffusion sensitization followed an HCP‐like dMRI protocol with three *b* values, 1000, 2000, 3000 s/mm^2^, and 90 directions per *b* value^145^ and identical preprocessing was applied to both datasets.^149^ The former dataset represented a high spatial‐ and angular‐resolution scan, and the latter dataset a low spatial‐ and high angular‐resolution counterpart. A subset of the 2.5 mm dataset with 60 uniform directions for *b* = 2000 s/mm^2^ was used to represent low spatial and low angular resolution. Up to three fiber orientations were estimated in each voxel of each dataset using the generalized ball and stick model.^141^ A tractography protocol defined in MNI space was then used (left panel), including a seed region at the hand area of the motor cortex (red) and two axial waypoint masks at the internal capsule (blue). The modes of the path distributions obtained using probabilistic tractography are shown for each case (results thresholded at path probabilities of 0.5%) in axial and sagittal views. The four arrows correspond from medial to lateral to: cortico‐thalamic, cortico‐bulbar, cortico‐spinal and cortico‐striatal projections

In Figure 4, we illustrate a simple example of how changing spatial and angular resolution of the data affects tractography. In this particular case, the increased spatial resolution is beneficial in differentiating thin projections from the hand area of the motor cortex. Apart from better resolving partial volume, higher spatial resolution is expected to increase the estimation accuracy of relatively short paths, which comprise the majority of brain connections.^19^, ^133^ It is important to note, however, that increasing spatial resolution at the expense of contrast‐to‐noise ratio or angular resolution can actually lead to suboptimal performance,^146^, ^150^ particularly in tracking major bundles.

Deciding on the acquisition protocol and getting the balance of these features right is governed by a series of trade‐offs (for instance SNR competing against spatial resolution or angular contrast). As shown in Figure 4, it may not be optimal if a feature is improved at the expense of others. Recent frameworks attempt to resolve these trade‐offs by fusing complementary datasets (e.g. data with high spatial and low angular resolution with data with low spatial and high angular resolution).^147^, ^151^ Another group of post‐processing methods boost certain features to improve estimation. These include denoising approaches for improving SNR^152^, ^153^ and up‐sampling or super‐resolution methods for improving spatial/angular resolution.^154^, ^155^, ^156^ Nevertheless, we can expect recent technological advances to provide better operating points for all these competing features and improve overall data quality. Hardware developments in modern scanners,^157^ such as higher gradient strengths, can translate to improvements in SNR and contrast of routine scans. While higher field strengths might pose difficulties to dMRI acquisitions due to the shorter *T*_2_ relaxation times at high field,^157^ they can be used to achieve very high spatial resolution.^143^, ^158^

Sequence developments should accompany these hardware advances. For instance, simultaneous multislice or multiband acquisitions^159^, ^160^ allow three‐ to fivefold acceleration of dMRI scan time, changing the perception of the data quality that can be achieved in realistic time frames. Faster scan times translate to higher spatial and/or angular resolution and/or SNR per unit time. Diffusion sensitization using double‐pulsed field gradients (see Shemesh et al^161^ for a review) or generalized trajectory imaging^162^ open new possibilities in probing restricted compartments and microscopic features with the potential to improve the accuracy of mapping from diffusion measurements to tissue structure.

Better data enable improved modeling and analysis.^60^ In fact, new tools are required to take full advantage of the new information in certain applications. An example is increasing spatial resolution in the presence of inevitable subject motion and eddy currents. The higher the resolution, the higher the need is for accuracy in distortion correction tools. New frameworks in this area^163^, ^164^ have been shown to limit alignment errors between dMRI volumes to less than a quarter of the voxel size^165^ and improve distortion correction,^166^ hence preserving the benefits of high‐resolution acquisitions after preprocessing.

## 3 VALIDATION AND COMPARISON WITH OTHER MODALITIES

We have highlighted a series of limitations for mapping the connectome using dMRI. It is therefore important to quantify the effect of these limitations on the ability to accurately map connectomes. It is also important to explore the utility and biological consistency of current connectome mappings given the limitations of dMRI and tractography and that an increasing number of frameworks are being developed with this aim (e.g. References^78^, ^97^, ^167^, ^168^, ^169^).

In the following sections we review studies that specifically compare tractography‐induced estimates with estimates from other modalities; either directly for the purpose of validation or indirectly for the purpose of multi‐modal integration. The comparisons show differences (and limitations as highlighted in the previous sections), but also provide evidence of agreement and predictive power in various different contexts that are greater than expected due to chance.

### 3.1 Direct evidence

There have been efforts to directly validate parts and aspects of the tractography‐estimated connectome with different invasive modalities, such as chemical tracers, primarily in animals. Tracers have their own limitations and biases.^17^ For instance, identifying correspondence between injection sites and a particular brain area is not straightforward. Axons that traverse the injection site can result in the reconstruction of spurious connections because these axons can absorb the tracer even though they make no synaptic contacts with the injection site (particularly in the rat brain, where fibers can bifurcate in gray matter). Absolute quantification of connection strength is also difficult, as anterograde and retrograde tracers depict different features of connectivity. However, tracers are very precise in spatial localization and have a considerably lower false positive rate than tractography. Thus, even if not perfect ground truths, they are much closer to the ground truth than *in vivo* dMRI.

Validation efforts have focused on the ability of tractography to estimate the existence of edges (and the route of underlying connections), as well as the relative strength of edges (i.e. treating the connectome as a binary or weighted matrix, respectively). The main conclusions that can be drawn are the following. (i) Tractography predictions are above chance; however, features with poor agreement exist. (ii) There is a trade‐off between sensitivity and specificity. Tractography methods that tend to be more sensitive in finding connections are also less specific. (iii) Cortico‐subcortical, short‐range intra‐hemispheric and homotopic inter‐hemispheric connections are more reliably estimated. (iv) Weights estimated by tractography provide a reasonable estimation of connectivity strength for some pairs of regions. (v) Parcellated connectomes, i.e. those estimated with nodes corresponding to mid‐scale regions, are more accurate than denser ones that attempt to depict fine, within‐region, details. In the following paragraphs we review the relevant studies and findings in more detail.

#### 3.1.1 Identifying connection trajectories

The first validation studies investigated how well tractography can identify large bundles and follow them through white matter. Qualitative comparisons have shown good agreement against histological tracing in the monkey brain^170^ or against dissected human samples.^171^, ^172^ Newer dissection methods^173^ allow preservation of the cortex and of the superficial white matter and have shown examples of the ability of tractography to follow the true route of connections up to their terminations.^174^, ^175^ However, semi‐quantitative comparisons have also highlighted false positive connections that are estimated.^176^, ^177^

**Figure 5:**
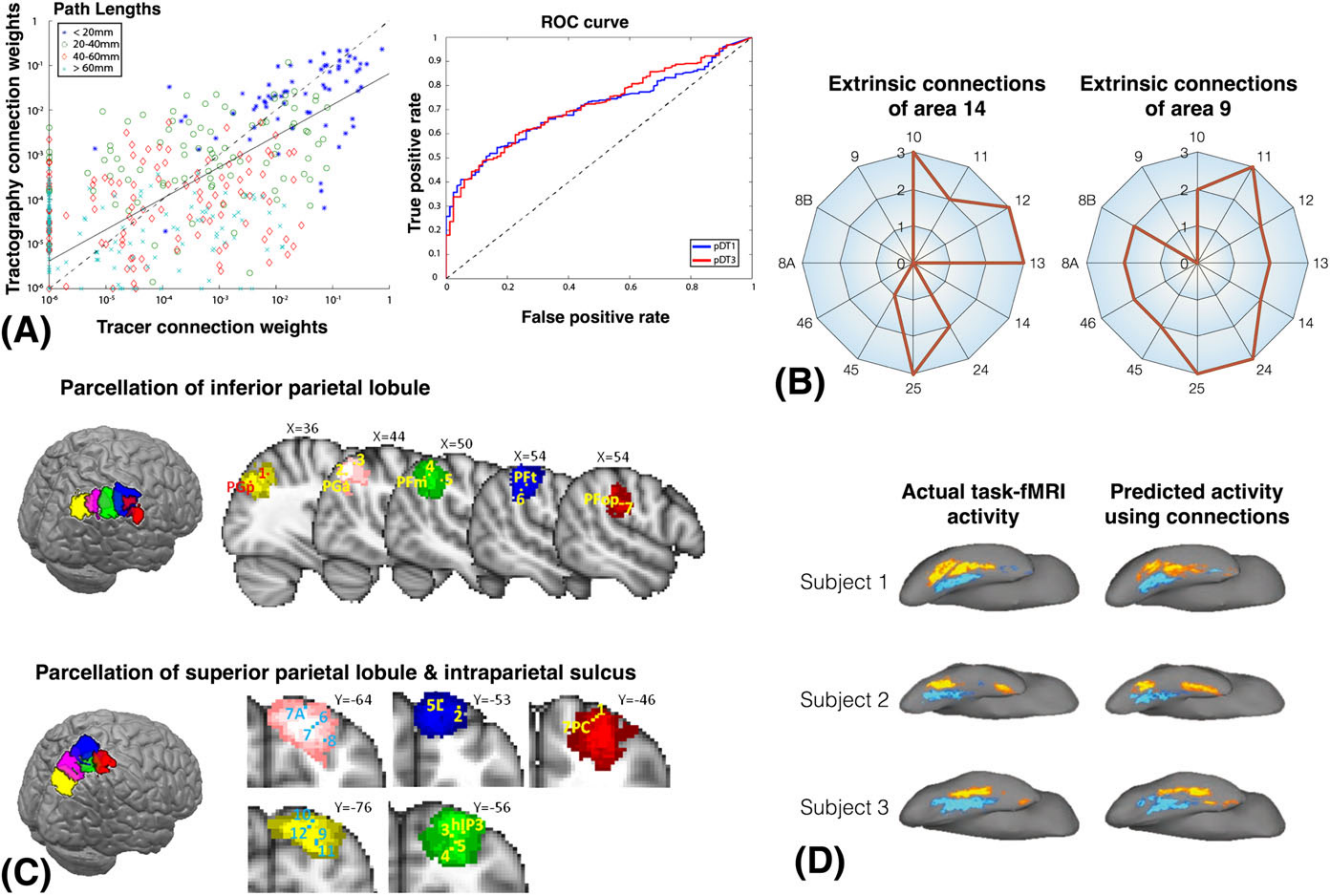
A, Correlation between connection weights inferred from tracers and tractography in the macaque connectome (correlation coefficient *r* ~ 0.55).^23^ The correlation is primarily driven by shorter connections. ROC curves for two alternative tractography algorithms, as benchmarked against a ground truth derived from tracers. An area under the curve of about 0.72 suggests that tractography does better than chance, but it is far from perfect. B, Connectivity fingerprints of two functionally distinct prefrontal areas, as measured using tracers in the macaque brain and presented in Passingham et al^4^. The radial distance represents connection strength (1, weak; 2, medium/ambiguous strength; 3, strong). C, Two parcellations of the parietal cortex based on the “connectivity fingerprints” methodology, as estimated by *in vivo* tractography in humans.^44^ For each case, sagittal (or coronal) views of the parcels are shown superimposed with the centre of gravity of cytoarchitectonic regions and center of mass of fMRI activations in various relevant tasks. The functional relevance of these parcels is demonstrated by the marked overlap. D, Predicting activity in the face area of the fusiform gyrus with a model that uses only the pattern of extrinsic connections of the fusiform gyrus to the rest of the brain.^5^ The actual activity measured using task fMRI is also shown for comparison. Notice how individual variability in activation during task is predicted by the respective individual variability in the connections. All panels are reproduced with permission

The sensitivity (finding true connections) and specificity (avoiding false connections) of tractography in detecting connectome edges has been more systematically explored in comparison to chemical tract tracing in the monkey,^23^, ^90^, ^178^, ^179^ mouse^55^, ^56^ or porcine brain.^180^ Areas under the curve in receiver‐operating characteristic (ROC) plots have been reported in the range of 0.7 to 0.8, suggesting a fair estimation accuracy in identifying connections and/or their routes (Figure 5A). At the same time, all studies illustrate the strong dependence of the results on the particular tractography settings and models. Probabilistic tractography is less sensitive than deterministic to anisotropy and curvature thresholds or the tissue composition of the seed.^178^, ^179^ It also tends to be more sensitive, but less specific, than deterministic methods and more susceptible to false positives.^97^, ^178^, ^180^ In general, an increase in sensitivity for all methods comes at the expense of a decrease in specificity, and therefore an optimized set of parameters is important. Also, tractography performs much better when exploring connections between relatively large cortical nodes rather than fine details within regions.

Comparisons of dMRI‐estimated paths with tracers also reveal benefits of considering fiber crossings in tracking (larger benefits are shown in some cases^180^ than in others^178^). Adding prior knowledge to guide connectome mapping is also beneficial.^56^ In Jbabdi et al^181^, organizational principles of cortical projections identified with tracers were found and generalized to post‐mortem macaque MRI data using informed tractography protocols that included *a priori* specified waypoint and exclusion masks. These were then used to search for and identify similar principles in humans. In Draganski et al^182^, subcortical connectivity signatures were obtained and revealed a series of networks between basal ganglia sub‐nuclei and the cortex. Some of these networks, in particular cortico‐striatal circuits, were directly validated against previous tracing studies in monkeys.

#### 3.1.2 Tractography‐estimated weights

An even more challenging task than localizing connections is extracting relative weights for the connectome edges. The recent development of comprehensive and weighted brain mappings by collating a plethora of tracing experiments^19^, ^20^ has permitted direct comparison with tractography‐derived connectomes. From these studies, we can extract some general features of the connectivity weights as measured by tracers: (i) There is a wide range of connectivity strengths than spans five orders of magnitude. (ii) An exponential reduction of connectivity strength occurs with path length.^133^, ^134^ (iii) There is a prevalence of local connections. Short connections are significantly more common and significantly stronger than long connections.

In one of the most recent comparisons, the authors evaluated the accuracy of dMRI connectomes in predicting generic features extracted from human brain dissections.^102^ The connectome edges and weights correctly replicated the relative percentage of long inter‐hemispheric and intra‐hemispheric connections and the inverse relationship between connection strength and length.

Non‐human primate studies allow more detailed comparisons due to the plethora of available ground truth. Donahue et al^23^ compared the connectome edges and weights obtained via macaque tractography and macaque tracers. Tractography could recover four out of the five orders of magnitude in the range of connection strengths. The performance was far from perfect, yet better than chance, even for the weakest of long paths (Figure 5A). Strong and short connections were better characterized by tractography, as the performance worsens with smaller connection weights. Overall a correlation of about 0.55 §
To put these correlation values into perspective, comparisons between histology and MRI‐derived values of a much more straightforward feature, such as cortical thickness, give correlations of the order of 0.6–0.7,^183^, ^184^ reflecting the inter‐modality variability and difficulty of direct comparisons. was reported between edge weights in tracer and tractography, and the path length dependence contributed significantly to this correlation. Interestingly, however, after regressing out path lengths, there was still a significant correlation (but smaller ~0.25) between the two measures, showing that it is not only the path length dependence that drives the relationship. Similar trends were reported by Van den Heuvel^124^ (though with smaller overall correlations, ~0.35, and a different definition of connectome weights, focusing on absolute streamline counts rather than relative contrasts of weights).

The dependence of these correlations to the node size was explored in References 55, 56 and for the mouse brain. Connection weights between mid‐level sized regions were more reliable (correlation up to 0.77 in Calabrese^56^) compared with considering small nodes (correlation dropped to 0.45). Comparable correlations were observed for the monkey brain.^57^ As discussed in the previous sections, certain limitations reduce the reliability of tractography at finer scales. Despite the errors, all the validation studies provide evidence that dMRI‐induced connectomes contain relevant information and predictive power. Interpretation might not always be straightforward, but a large number of applications show that this information can be useful. We review some of these applications in the following section.

### 3.2 Indirect evidence

The idea of connectivity fingerprinting using tractography weights (Figure 5B)^4^ has been applied to identify boundaries of functionally distinct regions (see References 45, 185 for reviews). In one of the earliest studies,^43^ a dense weighted matrix mapping connections from the medial frontal cortex to the rest of the brain was obtained using tractography. This matrix was used to identify the boundary between the anterior and posterior parts of the supplementary motor area. These are functionally distinct areas in both motor and cognitive domains, and the boundary, identified as a sharp change in the tractography profiles, agreed well with that obtained from functional MRI localizer tasks. Since then, similar results in predicting functional boundaries using dMRI and agreement with functional MRI have been shown for various cortical^41^, ^44^, ^186^, ^187^, ^188^, ^189^, ^190^, ^191^, ^192^ and subcortical areas^42^, ^193^, ^194^, ^195^ (see Figure 5C). Agreements have been also illustrated with other non‐MRI functional measurements, including PET^120^ and direct electrophysiological recordings,^196^ as well as cytoarchitectonic delineations.^197^, ^198^

Tractography “signatures” are reproducible and robust across subjects to a degree that allows predictions for the efficacy of targets in functional neurosurgery.^199^, ^200^ In deep brain stimulation, neurosurgeons search for the most efficient stimulation target via trial and error. Pouratian et al^201^ identified in this classical way the thalamic location that when targeted for stimulation allowed the most efficient alleviation of tremor symptoms. They then estimated using previously acquired dMRI the connectivity fingerprint of that region. New patients were scanned prior to surgery and the thalamic region best matching this fingerprint was identified and used as the initial target for stimulation. They found that in these new patients the tractography‐informed target was indeed very close to the most efficacious location, allowing better surgical planning.

The functional relevance of structural connectomes has been further illustrated by studies that use structure to constrain or predict function (Figure 5D). Models of functional networks built on a structural backbone have increased explanatory power and identifiability compared with models that lack such structure.^202^, ^203^ Structural connectivity networks alone have been used to predict the existence, strength and spatial features of functional connections as assessed by fMRI at rest.^204^ During a task, the functional activation maps have been predicted solely by the connection pattern of the activated region.^5^ More specifically, Saygin et al^5^ learnt a model in a group of subjects between the tractography‐estimated connectome of the fusiform gyrus and the functional activity recorded via fMRI during a face selection task. They then applied that model to the connectome of new subjects and predicted their task activation. This predicted activation was found to be very similar to the activation individually measured by subsequent fMRI. A subsequent study used a similar mapping approach and predicted the functional organization in young children after they learnt how to read, using the structural connection patterns they had developed prior to acquiring this skill (i.e. a few years earlier in their development).^205^ Such structure–function relationships can be observed even at a higher level, with structural connections predicting behavior and decision‐making processes.^206^, ^207^, ^208^

Mapping connection patterns offers the unique ability to perform comparative anatomy (see Mars et al^209^ for a review). This allows the identification of “homologue” areas across species (areas that share similar “connectivity contrast”) and the translation of the vast literature of animal studies to humans,^210^, ^211^, ^212^, ^213^ but also translation from humans to other primates to study evolution.^214^, ^215^, ^216^, ^217^ Such studies provide evidence of agreement of the structural estimates with a large range of independent sources of information.

## 4 NETWORK ANALYSIS

**Figure 6:**
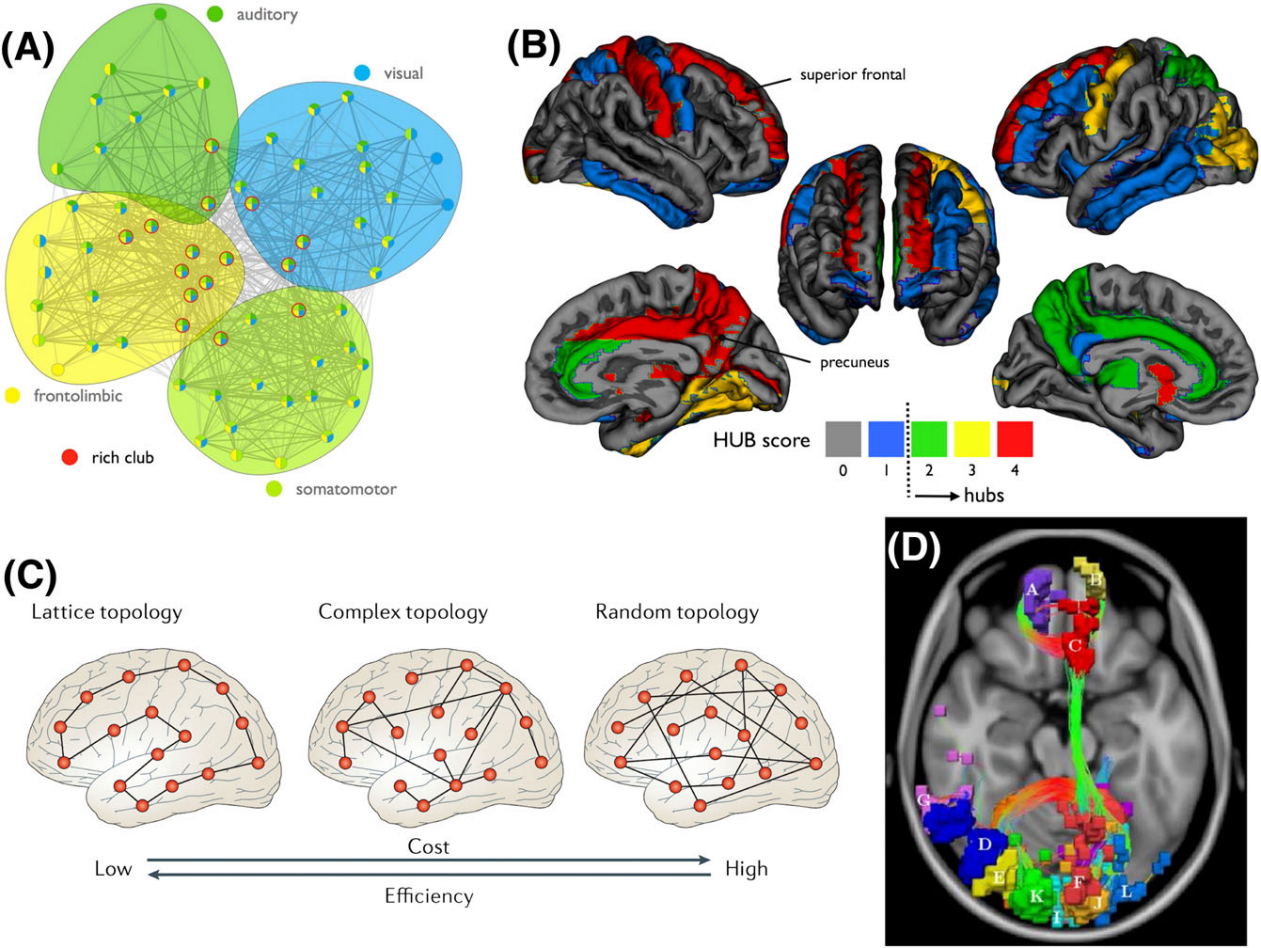
Characteristic network properties of brain graphs. A, Brain graphs are modular networks. In this example, each of the four modules is encapsulated by a distinctly colored bubble and comprises a densely interconnected set of cortical regions that perform a specialized function. The four modules are interconnected by a network of hub nodes (outlined in red) that form a rich club. Cortical regions in distinct modules most often communicate via routes traversing the rich club. The modules shown have been delineated in the cat connectome reconstructed with tract tracing. B, Hub nodes of the human connectome. Hub scores represent consistency across measures of node degree and centrality. Hub nodes are convergence and divergence points of neural information. C, Network economy refers to the trade‐off between network cost and network efficiency. Brain graphs are complex networks that attain a trade‐off between the low cost of lattice networks and the high efficiency of random networks. The addition of a small number of long‐distance connections to a lattice network results in a small increase in network cost, but a substantial reduction in the average number of connections that need to be traversed to establish a route between pairs of nodes. D, Network of disrupted connections comprising significantly fewer streamlines in patients with schizophrenia compared with healthy individuals. Each of the colored regions represents a node. Streamlines are only shown for disrupted connections. The cingulum bundle, genu and splenium of the corpus callosum can be seen to be disrupted. A reproduced from De Reus et al^218^, B from Van den Heuvel et al^128^, C from Bullmore et al^219^ and D from Zalesky et al^220^ with permission

Connectivity fingerprinting approaches discussed in the previous section (Figure 5B,C) mostly reflect hypothesis‐driven analyses, where a subset of the connectome is considered and relative connection patterns associated with certain nodes are examined. An alternative approach considers network properties, either in a data‐driven manner or with respect to hypothesis‐driven regions. Complex networks have been studied in mathematics for centuries, where they are known as graphs and are fully defined by their nodes and edges (Figures 1 and 6). In this section, we consider how graph theory and network science can be used to understand the global organization of the connectome.

The edges of a graph are either directed or undirected. Edges inferred from tractography are invariably undirected because the direction of diffusion cannot be resolved with dMRI (in contrast to tract tracing methods, which can distinguish between afferent and efferent fibers). Furthermore, the edges of the graph can be either weighted or binary, as previously discussed.

### 4.1 Adjusting density and weights

Thresholding methods applied to brain graphs with weighted edges reduce the density of connections in a graph and aim to eliminate spurious edges, thus improving specificity. Thresholding can be further applied to binarize graphs^221^, ^222^ and simplify the interpretability of certain analyses by emphasizing network properties that may be obscured by large variations in edge weights. On the other hand, thresholding may disregard useful information (see previous section and representative examples in References 5, 43, 206). Therefore, the applicability of such approaches depends on the particular question of interest.

The simplest thresholding method, called *weight‐based thresholding,* involves eliminating any edges with a weight that is below a given global threshold. To yield a binary graph, the weights associated with the remaining edges are disregarded, leaving only information about whether edges are absent (0 in connectivity matrix) or present (1 in connectivity matrix). Weight‐based thresholding introduces the confound of graph density to comparisons between groups of individuals. Graph density ¶
The term “connection density” is found in the relevant literature. We use “graph density” here to avoid confusion with connection axonal density that we refer to in other sections of the paper. —the proportion of all node pairs that are directly interconnected by an edge— fundamentally influences the properties of a graph.^222^ Applying the same global threshold to different brain graphs does not necessarily ensure that the resulting thresholded graphs have the same density. Therefore, when complex properties of thresholded brain graphs are found to differ between individuals, it is unclear whether these differences are trivially due to differences in graph density.

*Density‐based thresholding* 222 overcomes this confound. A unique threshold is determined for each individual to ensure a fixed graph density for all individuals. The disadvantage of this approach is that the number of spurious connections may differ between individuals because different absolute thresholds are used, which introduces a new confound.

The differences between thresholding methods are evident when comparing brain graphs between different groups (e.g. patients and healthy controls).^221^ For instance, many white matter connections in patients with schizophrenia comprise significantly fewer streamlines when compared with healthy individuals.^220^ Therefore, density‐based thresholding is likely to result in brain graphs that comprise more spurious connections in patients compared with controls.^223^ This may explain the subtle randomization of network organization reported in patients with schizophrenia.^220^, ^224^ In particular, density‐based thresholding may force the inclusion of spurious edges into the patient brain graphs, leading to randomization. On the other hand, if weight‐based thresholding is used, it is important to recognize that potential differences in complex network properties may simply reflect a lower overall number of connections in one group. Ultimately, the distinction between these two thresholding methods boils down to whether differences in complex network properties should be divorced from differences in graph density.

A variety of alternative thresholding methods have been developed to preserve or emphasize specific features of brain graphs. Fragmentation of a graph into disconnected islands of nodes is undesirable and anatomically unrealistic. To avoid fragmentation, a *minimum spanning tree* can be formed based on the edges with the highest weights.^225^ By definition, this yields a connected graph in which paths can be found between all node pairs. Further edges can then be progressively added to the minimum spanning tree until a desired graph density is achieved. Local thresholding methods attempt to preserve graph structures that span multiple scales of edge weights. Whereas global thresholding is invariably based on a single threshold, local thresholding methods such as the disparity filter^226^ seek to calculate distinct thresholds for each node and its associated connections. Finally, thresholding can be performed to preserve edges that are consistently found between individuals in a group.^227^ Such consistency‐based thresholding can however eliminate connections that genuinely vary between individuals.

While thresholding is not an essential prerequisite to brain graph analysis (see for instance the connectivity fingerprinting examples described in Section 3 and in Figure 5), it is often performed to: (i) improve the interpretation of topological descriptors; (ii) ease computational and storage burden; (iii) control for the effect of between‐group differences in graph density when performing between‐group comparisons of network properties and (iv) minimize the number of spurious (false positive) connections. Including spurious connections (false positives) is substantially more detrimental to the topological analysis of brain graphs than failing to detect genuine connections (false negatives), and thus thresholding is considered a crucial step to maximize the specificity of brain graphs.^96^ The choice of thresholding method (if any) should be guided by the requirements of subsequent analyses. For example, thresholding methods that yield binary graphs are inappropriate if the goal is to test for between‐group differences in the weights associated with each edge.

### 4.2 Network properties of brain graphs

Many intriguing properties of brain graphs have been discovered during the last decade. These properties are not unique to the human brain and are often found universally across many species and imaging scales. Brain graphs are *small‐world* networks that are *modular* in structure.^228^ Modular small‐world networks are characterized by communities of nodes that are densely interconnected among themselves, predominantly with relatively short connections, while also sparsely connected to other communities by a small number of long‐distance connections (Figure 6A). These densely interconnected communities (modules) are hypothesized to facilitate network segregation and specialized information processing. The longer connections interconnecting these modules facilitate network integration and distributed information processing. Modules in brain graphs tend to be spatially localized and comprise cortical regions that perform specialized functions, such as visual, auditory or motor processing tasks.

Brain graphs also comprise a densely interconnected core of *hub nodes* that form a *rich club*.^229^ Hub nodes make connections with many other nodes and serve as focal points for the divergence and convergence of neural information. Hubs are defined as nodes with large degrees, where the *degree* of a node is simply the total number of other nodes to which it is directly connected.^230^ The nodes of brain graphs differ very substantially in degree. Indeed, the distribution of nodal degrees in most brain graphs can be described by a truncated power law (scale‐free distribution), which implies the existence of a small number of highly connected hub nodes. For a hub node to form part of the rich club, it must also be densely connected with other rich‐club nodes. A rich club in general comprises nodes (hubs or non‐hubs) that are densely connected among themselves as well as with other nodes. Non‐hub nodes of the club are called peripheral or local nodes. Rich clubs are synonymous with many kinds of natural and engineered network. For example, major air transportation hubs are interconnected to an extent that is significantly greater than expected in random networks with the same nodal degree distribution. While the hub nodes comprising a rich club are usually distributed across different modules, they are also densely interconnected by long‐range connections. This suggests that the rich club is a network core that plays a crucial role in integrating and coordinating the activities of specialized modules. Typical hub nodes comprising the rich club of brain graphs include the caudate, thalamus, precuneus, superior frontal gyrus and middle cingulate gyrus (Figure 6B).

Another property of brain graphs is *network economy*.^219^ Brain graphs are spatially embedded networks in which each node is associated with a coordinate in Euclidean space and each edge has a physical length.^135^ Long connections are considered costly to nervous systems in the sense that they occupy more physical space and consume more metabolic resources.^231^ A small number of long‐distance connections is however crucial to ensure that information can be efficiently integrated between different modules. Spatially embedded networks that comprise only short connections are not very costly in terms of their energy and space requirements, but they are also not very efficient integrators of information. Indeed, spatially embedded networks comprising only short connections are lattice‐like networks in which distant pairs of nodes must traverse many connections to communicate. The number of connections that need to be traversed, known as the *path length*, can be drastically reduced with the addition of a small number of long‐distance connections.^232^ Network economy refers to this trade‐off between the cost of a network's topology and the efficiency with which that network can integrate information (Figure 6C). In practice, efficiency is quantified as the inverse of the path length between all node pairs,^233^ while cost is quantified as the sum of the physical length of all connections. Numerous studies indicate that nervous systems have evolved to negotiate a compromise between network efficiency and network cost.^234^

### 4.3 Comparing network properties between groups

Comparing the network properties of brain graphs between groups of individuals can reveal new insights into brain network organization in health and disease (see Griffa et al^10^ for an extensive review). Statistical inference can be performed on the connectome at many different scales. The simplest data‐driven approach is to independently test the weights associated with each edge for a between‐group difference or a statistical association with some measure of cognitive performance. Network‐specific methods can be used to identify between‐group differences given the network structure and correct for multiple comparisons. The network‐based statistic^235^, ^236^ is an example of one such non‐parametric approach that identifies interconnected subnetworks for which the null hypothesis (of no differences between groups or no associations with chosen score) can be rejected. Given that brain pathology seldom impacts a single connection in isolation,^237^ these mass univariate approaches aim to identify multiple network elements that significantly differ in connectivity strength between groups. In many brain diseases, white matter connections provide conduits that promote the spread and spatial propagation of a pathological mechanism,^238^, ^239^ and thus it is not surprising that disease‐related connectivity effects often form interconnected subnetworks (Figure 6D).

Another approach to statistical inference is the testing of between‐group differences in global summary measures of network organization, such as measures of network efficiency,^219^ small‐worldness^240^ and modularity.^241^ Global testing is simple but lacks specificity, in that insight cannot be gained into whether effects are distributed throughout the brain or confined to a specific set of nodes or edges. Finally, multivariate statistical inference is growing in popularity and may play an important role in the future as datasets continue to increase in size and complexity. Multivariate approaches seek to learn complex patterns among multiple elements of a brain graph and utilize these patterns for inferential classification and prediction. Support vector machines,^242^ partial least squares^243^ and canonical correlation analysis^244^ have been successfully applied to connectomic data.

Interpreting clinical differences in brain graphs can be challenging. These can be further complicated due to the complex patterns of epiphenomena and adaptive responses, as well as the progressive nature of connectivity deficits in many brain diseases. In general, analysis of brain graphs in clinical populations requires care in the choice of network measures analyzed—testing for between‐group differences in a large set of arbitrarily chosen properties of a brain graph is unlikely to be fruitful. Ascribing biological meaning to some network measures is also challenging and based on many assumptions. For example, measures of path length tacitly assume that neural information is transmitted via the shortest paths in a brain graph,^219^, ^231^ but this remains to be established, and other models of transmission based on diffusion processes and greedy navigation may be considered more plausible.^245^

## 5 SUMMARY

Using dMRI to map macro‐connectomes *in vivo* has shown promise in exploring brain organization and advancing our knowledge of brain connection patterns and network properties that are difficult to elucidate with alternative techniques.

However, mapping connectomes with dMRI remains challenging and caution is needed to ensure that connectomic analyses do not demand or assume fiber reconstruction accuracies that stretch beyond inherent limitations of macroscale imaging modalities. It is important for investigators to be aware of these existing limitations that affect the applicability, accuracy and interpretation of connectome reconstructions. Open problems in the various stages of building connectomes set new interesting and challenging questions. They provide new opportunities for acquisition and methodological developments, which are necessary to progress the field. Validation studies based on microscopy, tracers and histology are also needed. Such comparisons can assist with identifying the major challenges and modes of failure in a systematic way and motivate new developments.

Multimodal imaging and analysis may overcome some of the limitations of dMRI. Apart from using dMRI, brain networks can be also estimated using anatomical,^246^ resting‐state functional^247^ and task‐based functional MRI,^248^ while temporal dynamics can be estimated using MEG^249^ and EEG.^250^ Since all these approaches are indirect, each is accompanied by its own assumptions, limitations and independent sources of errors.^17^ Therefore, having multiple windows to the true physical connectivity can filter out inaccuracies inherent to particular modalities and provide stronger support for the reliability of the findings.^148^ This cross‐modal paradigm has been used by large initiatives, for instance the Human Connectome Project,^121^, ^251^ and has proved beneficial in demanding problems.^39^

Currently, estimated parcellated connectomes with nodes corresponding to relatively large regions are more robust and reproducible, but they are also more likely to overlook detailed patterns of connectivity. Dense parcellations have proven useful when parts of the connectome are considered in a controlled, hypothesis‐driven analysis. However, they can potentially introduce difficulties in estimation when the connectome as a whole is to be used in an exploratory fashion. In the future, we can expect that solving some of the open problems will increase the accuracy of the different representations.
